# Comparison of SARS-CoV-2 whole genome sequencing using tiled amplicon enrichment and bait hybridization

**DOI:** 10.1038/s41598-023-33168-1

**Published:** 2023-04-20

**Authors:** Anita Koskela von Sydow, Carl Mårten Lindqvist, Naveed Asghar, Magnus Johansson, Martin Sundqvist, Paula Mölling, Bianca Stenmark

**Affiliations:** 1 Department of Laboratory Medicine, Clinical Pathology and Genetics, Faculty of Medicine and Health, Örebro University, Örebro, Sweden; 2 School of Medical Sciences, Faculty of Medicine and Health, Örebro University, Örebro, Sweden; 3 Clinical Genomics, Science for Life Laboratory, Faculty of Medicine and Health, Örebro University, Örebro, Sweden; 4 Department of Laboratory Medicine, Clinical Microbiology, Faculty of Medicine and Health, Örebro University, Örebro, Sweden

**Keywords:** Microbiology, Virology

## Abstract

The severe acute respiratory syndrome coronavirus 2 (SARS‑CoV‑2) pandemic has led to extensive virological monitoring by whole genome sequencing (WGS). Investigating the advantages and limitations of different protocols is key when conducting population-level WGS. SARS-CoV-2 positive samples with Ct values of 14–30 were run using three different protocols: the Twist Bioscience SARS‑CoV‑2 protocol with bait hybridization enrichment sequenced with Illumina, and two tiled amplicon enrichment protocols, ARTIC V3 and Midnight, sequenced with Illumina and Oxford Nanopore Technologies, respectively. Twist resulted in better coverage uniformity and coverage of the entire genome, but has several drawbacks: high human contamination, laborious workflow, high cost, and variation between batches. The ARTIC and Midnight protocol produced an even coverage across samples, and almost all reads were mapped to the SARS-CoV-2 reference. ARTIC and Midnight represent robust, cost-effective, and highly scalable methods that are appropriate in a clinical environment. Lineage designations were uniform across methods, representing the dominant lineages in Sweden during the period of collection. This study provides insights into methodological differences in SARS‑CoV‑2 sequencing and guidance in selecting suitable methods for various purposes.

## Introduction

The rapidly spreading worldwide pandemic of severe acute respiratory syndrome coronavirus 2 (SARS‑CoV‑2) has led to extensive virological monitoring by whole genome sequencing (WGS). Genomic surveillance and characterization of the full-length SARS-CoV-2 sequence is necessary to understand its spread, transmission, and evolution. Virological monitoring has been performed in a joint global collaboration, with more than > 13 million SARS-CoV-2 whole genomes generated and shared on the GISAID platform as of September 2022. This genomic resource has supported public health decision-making throughout the COVID-19 pandemic, allowing detection of variants that might affect virulence, pathogenesis, and immune escape^1^. Similarly to other RNA viruses, SARS-CoV-2 evolves and changes continuously, giving rise to variants with different degrees of infectivity and lethality. Variants of concern have been classified by the World Health Organization as the Alpha, Beta, Gamma, Delta, and Omicron variants. The evolution of these variants includes accumulation of mutations in the spike protein in particular^2^.

To generate complete viral genome coverage at a low sequencing cost from clinical samples, without isolation or culture, target enrichment methods are often required^3^, since clinical samples contain predominantly human cells with only a minor proportion of virus. WGS of SARS-CoV-2 is mainly performed via multiplex PCR amplification or sequence hybridization by bait capture^4^. Sequencing using multiplex PCR amplicons is perhaps the most common approach for population-scale viral surveillance, due to its simplicity and low cost. However, new variants in primer binding sites may cause amplicon dropout or uneven sequencing coverage, resulting in loss of information or inaccurate data^5,6^. One of the most widely used amplicon methods is the ARTIC network protocol^7,8^. The protocol is flexible and exists in several versions, in which the PCR amplicons go through library preparation with sample-specific barcodes added, and are sequenced using either short-read (e.g. Illumina) or long-read (e.g. Oxford Nanopore, PacBio) technologies.

The Midnight protocol with multiplexed 1200 base pair PCR amplicons is a descendent of the ARTIC protocol customized for long-read sequencing using equipment from Oxford Nanopore Technologies (ONT)^9,10^. Long-read ONT sequencing offers rapid real time sequencing, with the advantages of portability, simple sample preparation, and scalability. ONT has been used for genome sequencing of infectious diseases of clinical samples, without isolation and culture, particularly for viral surveillance during the Ebola and Zika outbreaks^3,11^.

The hybrid capture-based method from Twist Biosciences relies on hybridization between a bait set (~ 120 bp) and the target sequence. The baits, which are made of biotinylated, single-stranded RNA probes complementary to the target DNA, can tolerate a 10–20% mismatch when comparing with the probe sequences^12^, which could be favourable given the emergence of new SARS-CoV-2 variants.

In this study, we aimed to compare three methods for SARS-CoV-2 WGS in a clinical setting: two tiled amplicon enrichment techniques and one bait hybridization technique, sequenced with Illumina and ONT, to provide insights into methodological differences between the methods.

## Methods

### Sample selection and extraction

A total of 47 clinical patient samples from swabs taken from the upper respiratory tract were obtained between 1 July 2020 and 1 April 2021. Total nucleic acid was extracted from heat-inactivated viral specimens by either the TANBead Nucleic Acid Extraction Kit on the TANBead Maelstrom 9600 (BioService, Sweden) or the MagDEA® Dx reagent for Nucleic Acid Extraction on the MagLEAD 12gC (Precision System Science Co, Tokyo, Japan). Extraction was performed according to the manufacturer’s instructions, with sample start volume of 200 µL eluted in 50 µL. A no template control (NTC) was included for each extraction batch. RNA extracts were stored at − 80 °C and kept on ice when thawed. An Allplex™ 2019-nCoV real-time PCR assay targeting the E, N, RdRp/S genes (Seegene Inc., Seoul, Korea) had been previously performed to identify SARS-CoV-2 in clinical routine at Örebro University Hospital, Sweden, as described elsewhere^13,14^. The samples included in the study had a RdRp/S gene Ct ranging from 14 to 30. The present study was a method evaluation of SARS-CoV-2 sequencing and all samples were collected according to regulations in routine diagnostics at Örebro University Hospital. Sequencing was performed using Clinical Genomics Örebro, Science for Life Laboratory infrastructure at Örebro University and Örebro University Hospital. We confirm that all experiments were performed in accordance with relevant guidelines and regulations. As the samples were from remaining material from routine diagnostics, de-identified and only used for a method evaluation study, ethical approval was not required according to the Swedish Biobank Act (2002:297) §2.

### Twist bait hybridization enrichment

The Twist Bioscience bait hybridization enrichment protocol for cDNA to library preparation and target enrichment was followed according to the instructions for the January 2021 version of SARS-CoV-2 NGS Assay-RUO (Twist Bioscience, San Francisco, CA). Briefly, the RNA was quantified using a Qubit Fluorometer and High Sensitivity RNA assay (Thermo Fisher Scientific). The manufacturer recommended 50 ng/samples, but the samples included ranged from non-quantifiable to 50 ng (35/47 samples were non-quantifiable using Qubit), and were included regardless of Ct values. cDNA was created, with the first strand synthesised using the ProtoScript II First Strand Synthesis kit with Random Primer 6, and the second synthesised using the NEBNext Ultra II Non-directional RNA Second Strand Synthesis Module (New England Biolabs, Ipswich, MA). Each sample of cDNA ≤ 25 ng was prepared with Twist’s unique dual indices by steps of fragmentation, end repair, and dA-tailing. Adapters were ligated and indexes were added to the amplified cDNA. Samples were multiplexed to a total mass of 1500 ng for target enrichment, and 8 amplified indexed libraries were pooled for each hybridization reaction, as per the manufacturer’s instructions. Capture of the hybridization reaction was performed for 16 h. The post-hybridisation capture was washed by magnetic Twist Streptavidin Binding Beads, and eluted in Streptavidin Binding Bead Slurry. The post-capture library was amplified and purified with Twist DNA purification beads. Paired-end sequencing was performed on the MiSeq with reagent kit v3 for 150 cycles (Illumina).

### ARTIC V3 tiled amplicon enrichment

The ARTIC V3 tiled amplicon enrichment protocol^15^ was used to create cDNA and to subsequently produce 400 bp tiled amplicons of the viral genome using ARTIC V3 nCov-2019 primer pools from Integrated DNA Technologies (Coralville, Iowa)^8^. The Quick protocol was followed aside from using the previous version’s SuperScript IV VILO master mix from Thermo Fisher Scientific (Waltham, MA). The cDNA was subsequently amplified, producing 400 bp amplicons. For each sample, two master mixes containing primer pool 1 or 2 were pooled and purified. Library preparation was performed with Illumina DNA Prep (Illumina, San Diego, CA) according to the manufacturer’s instructions. Samples were sequenced on the MiSeq (Illumina) with reagent kit v2 using 500 cycles with paired-end reads.

### Midnight tiled amplicon enrichment

Long-read sequencing libraries were generated from 1200 bp amplicons using the Midnight protocol based on the ARTIC network (ONT). The March 2021 protocol version for PCR tiled amplicon of SARS-CoV-2 virus with rapid barcoding (SQK-RBK110.96,^16^) was followed, except for usage of the V1 and V2 cDNA synthesis kit SuperScript IV VILO master mix (Thermo Fisher Scientific). Input viral RNA was equalized by Ct values, as described in the ARTIC V3 protocol, and extracted nucleic acid was reverse transcribed. cDNA was amplified using two SARS-Cov-2-Midnight-1200 primer pools (Integrated DNA Technologies, no. 10007184). For each sample, two multiplex PCR reactions were performed, containing primer pool 1 or 2. The two pools were combined into one tube for each sample before rapid barcodes were added. All samples were pooled together, multiplexing up to 48 samples per flow cell, and cleaned up using magnetic SPRI beads. Rapid adapter was added to 600–800 ng of the library, mixed, and incubated for 5 min. Sequencing buffer, loading beads, and DNA library were mixed and loaded into a primed SpotON Flow Cell on a GridION system sequencer (ONT, Oxford, UK), according to the manufacturer’s instructions. The flow cell was washed by Flow Cell Wash Kit (ONT) and reused for > 800 functional pores. MinION sequencing was run for 12 h to generate ~ 200,000 reads per sample using MinKNOW Software (ONT) and fast base calling.

### Data analysis

Fastq and Fast5 files were analysed using the gms-artic pipeline developed within the Genomic Medicine Sweden collaboration^17^ using SARS-CoV-2 from Wuhan-Hu-1 (GenBank: MN908947.3) as reference genome. This is an adaption of the established workflows (ncov2019-ARTIC-nf and ARTIC minion) developed by the ARTIC Network and Public Health Wales. In short, Illumina reads were trimmed with Trim Galore and aligned with BWA. iVar was used to trim the primer sequences and create a consensus sequence. For Nanopore reads, demultiplexing was performed with GuppyPlex and alignment with minimap2, and consensus was created with medaka (https://github.com/nanoporetech/medaka). Primer-scheme specific BED files were used to identify the regions of the mapped sequences corresponding to synthetic sequences (primers), and these regions were clipped to ensure that the sequences were entirely of biological origin. For both technologies, the quality of the run was evaluated with the Picard CollectAlignmentSummaryMetrics tool^18^.

The fold-80 base penalty was used to estimate uniformity. This metric describes how much more sequencing is required to bring 80% of the target bases to the mean coverage. It is ideally equal to 1, indicating an on-target rate of 100% and uniform coverage.

The capture enrichment efficiency of reads mapped to human, SARS-CoV-2, or not aligned was generated using BWA-MEM for Illumina data and minimap2 for nanopore data. Human reads were only counted and used for QC. Coverage at basepair resolution was created with SAMtools depth. Figures 2, 3, 4 and 5 were created in Rstudio version 1.2.5033 with R version 4.2.2 using packages ggplot2 3.4.0, reshape2 version 1.4.4, dplyr version 1.0.10, ggpubr version 0.5.0 and tidyverse version 1.3.2.

The consensus sequences were annotated for virus clade information using Nextclade (NEXTstrain version 1.14.1) and Pangolin (pangolin lineage version v3.1.20 with pangoLEARN version 2022-04-28). Consensus quality control (qc.overall and qc.missing) was extracted from the Nextclade output. The qc.overall metric was used to assess the overall quality of the consensus sequences, scores 0–29 are assigned as “good” quality, 30–99 as “mediocre” quality and ≥ 100 as “bad” quality. The qc.missing metric is based on the numbers of Ns in the consensus sequence, if the sequence misses more than 3000 sites (N characters), it will be flagged as bad. The first 300 missing sites are not penalized (https://docs.nextstrain.org/projects/nextclade/en/stable/user/algorithm/07-quality-control.html). The consensus sequences generated for each sample were compared using the whole genome alignment tool in CLC Genomics Workbench v 22.0 with default settings and the SARS-CoV-2 reference genome from Wuhan-Hu-1 (GenBank: MN908947.3) as reference. A multiple sequence alignment was extracted, and a phylogenetic tree was created and visualized using the Neighbor Joining tree algorithm in CLC Genomics Workbench.

All sequenced samples were reported to the Public Health Agency of Sweden for SARS-CoV-2 surveillance. Assembled FASTA consensus sequences are available at GenBank with submission-ID: SUB12123746 and accession-ID: OP585690-OP585783.

All methods were carried out in accordance with relevant guidelines and regulations.

## Results

**Figure 1:**
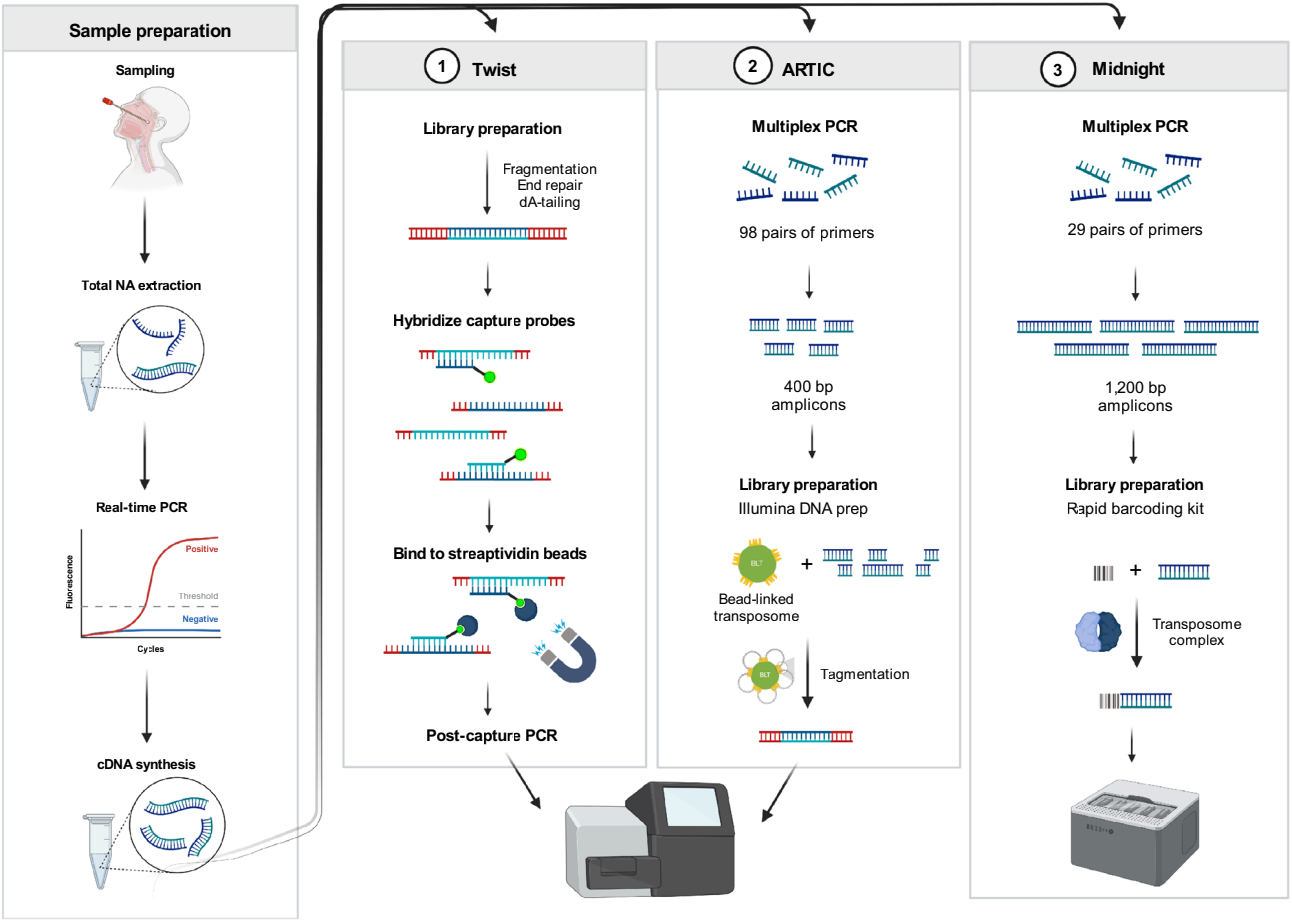
Schematic overview of the methods being compared. PCR-positive SARS-CoV-2 samples were sequenced with three different workflows; 1. Twist bait hybridization enrichment method sequenced on the Illumina MiSeq. 2. ARTIC V3 tiled amplicon enrichment sequenced on the Illumina MiSeq. 3. Midnight tiled amplicon enrichment long reads sequenced on the Oxford Nanopore Technologies GridIon. Image created by Bianca Stenmark with BioRender (BioRender.com).

A total of 47 SARS-CoV-2 positive samples with real-time PCR Ct values ranging from 14 to 30 were sequenced with three different protocols for SARS-CoV-2 sequencing, involving enrichment by bait hybridization or tiled amplicons. A schematic overview including library preparation, target enrichment, and sequencing is shown in Fig. 1. All 47 samples were run with Twist Bioscience bait hybridization enrichment, henceforth referred to as Twist, and ARTIC V3 tiled amplicon enrichment, henceforth referred to as ARTIC. For Midnight tiled amplicon enrichment, henceforth referred to as Midnight, only 24 of the original 47 samples were run because the rest of the RNA samples had been sent to external laboratories for national surveillance analyses or had been discarded. In the present study, Twist had an estimated time for library construction of around 38 h including 16 h hybridization time. The hybridization step can however be performed for 2 h instead of overnight for 16 h. ARTIC had an estimated time for library construction of approximately 9 h and Midnight approximately 7 h. Midnight library preparation had the shortest sequencing time set to run for 12 h, performed on the ONT GridIon. Sequencing time on the Illumina MiSeq was 21 h for Twist and 39 h for ARTIC. Data output was adjustable, and depended on the chosen sequencing platforms and kits.

### Comparison of mean depth and uniformity

**Figure 2:**
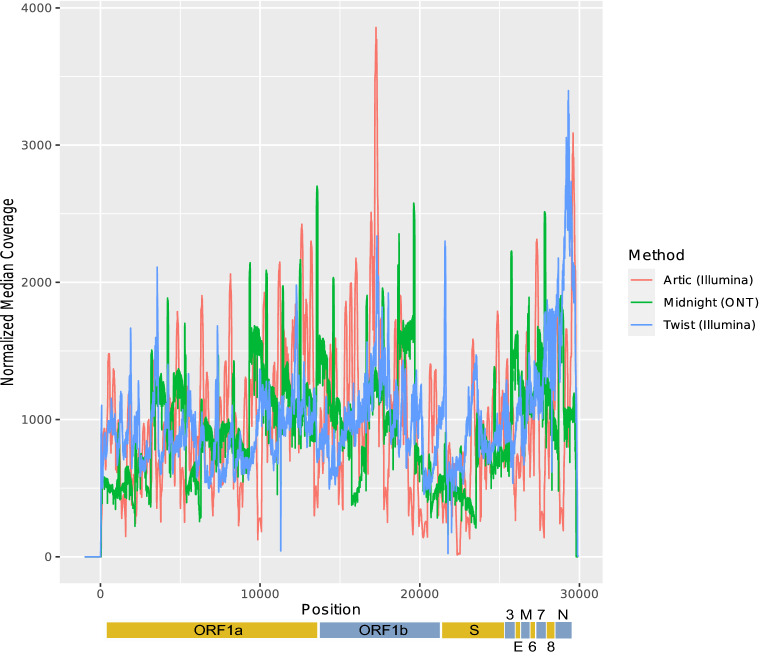
Mean sequencing coverage of the SARS-CoV-2 genome for each method. Sequencing depths are scattered along the SARS-CoV-2 genome, and the line for each method represents coverage of the raw medians for every position. Read coverage is normalized so that mean coverage is 1000 × for every sample. SARS-CoV-2 open reading frames (ORFs) are shown below the graph: 3, ORF3; 6, ORF6; 7, ORF7; 8, ORF8.

Sequencing metrics across methods are shown in Supplementary Table 1. Twist samples (n = 47) had the highest total reads, with a mean of 6,630,000 reads and median depth of 1,313 × (range: 4–60,104 ×). ARTIC samples (n = 47) had a mean of 745,000 total reads and a median depth of 2,724 × (range: 381–4,611 ×). Midnight samples (n = 24), had a mean of 160,000 total reads and median depth of 930 × (range: 12–3,322 ×). Excluding the diluted nucleic extractions for the Midnight protocol due to sample volume shortage (n = 7), the mean was 197,000 total reads and median depth 1440 × . The mean sequencing coverage depth of the SARS-CoV-2 genome, downsampled to 1000 × coverage for each method with raw medians, is shown in Fig. 2. Evenness was measured with the fold-80 base penalty, a metric to estimate uniformity which ideally is equal to 1. Using Twist, positions 11,288–11,296 and 21,766–21,770 had the lowest read depth coverage (23–45 × and 42–287 × , respectively). Twist had a fold-80 median of 1.47 (range: 1.22–2.4). ARTIC resulted in relatively low coverage in the open reading frame encoding the spike protein in position 22,339–22,523 (coverage < 23 ×); this might have been due to primer specificity and uneven amplification for all amplicons. ARTIC had a fold-80 base penalty median of 1.81 (range: 1.49–4.66). Peaks with high coverage were due to overlapping primer regions or improved amplicon genome recovery, seen in region 17 Mbp with ARTIC. Midnight exhibited relatively even coverage across all 29 amplicons, with lowest coverage in position 21,770 (161 ×) and 23,516 (209 ×) and a fold-80 base penalty median of 2.08 (range: 1.6–14.1).

### Comparison of viral load and genome coverage

**Figure 3:**
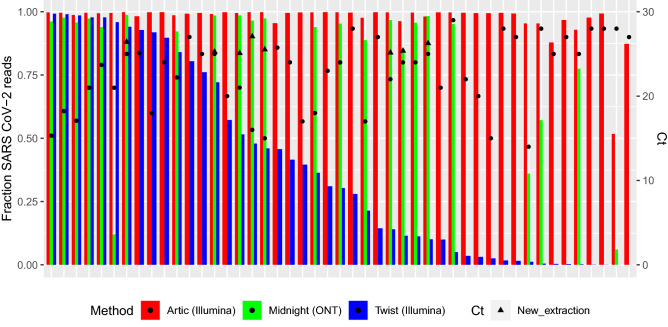
Performance of variation of mapped reads between methods for individual samples. Fractions of SARS CoV-2 mapped reads in stacks. Ct values are indicated by black dots, and samples are sorted by decreasing Twist fraction. Twist bait hybridization enrichment (n = 47 patients), ARTIC tiled amplicon enrichment (n = 47 patients), Midnight tiled amplicon enrichment (n = 24 patients).

The relations between reads mapped to SARS-CoV-2 and Ct values are shown in Fig. 3 and Supplementary Fig. 1. The first 10 samples in batches A–B of Twist were combined regardless of Ct values (Supplementary Fig. 1), leading to samples with high viral load (Ct < 18) having an excessively high amount of total reads (> 4.5 M). The rest of the Twist samples were sequenced with similar Ct range, which gave slightly less variation in the number of total reads and an even fraction distribution with a tendency toward a decreased fraction of SARS-CoV-2 at higher Ct values. In addition, 2/10 samples in batch A-B were unquantifiable, contradictory to 33/37 for batch C–G. No batch effect was seen in the ARTIC or Midnight samples (Supplementary Fig. 1), where the majority had a fraction > 0.75. The 24 Midnight samples were either from the same extraction used for the other methods (original extraction), from a dilution of the original extraction, or from a new extraction, as indicated in Supplementary Fig. 1.

### Intra-individual performance

Sequencing results revealed differences in the fraction of SARS-CoV-2 specific reads. Twist had a median of 30% of mapped reads (range: ≤ 0.001–99.7%), ARTIC had a median of 99.6% (range: 54.6–99.9%), and Midnight had a median of 94% (range: 7.8–96.8%); these differences were not related to viral load (Fig. 3). The fractions of SARS-CoV-2 reads found with different methods within the same patient are shown in Fig. 3. ARTIC had high fraction of SARS-CoV-2 mapped reads. Twist fractions dropped from 0.99 to 0.001, with lowest fractions seen for the same samples as ARTIC and Midnight (although at much higher fractions), which generally had high Ct values.

### Host content

**Figure 4:**
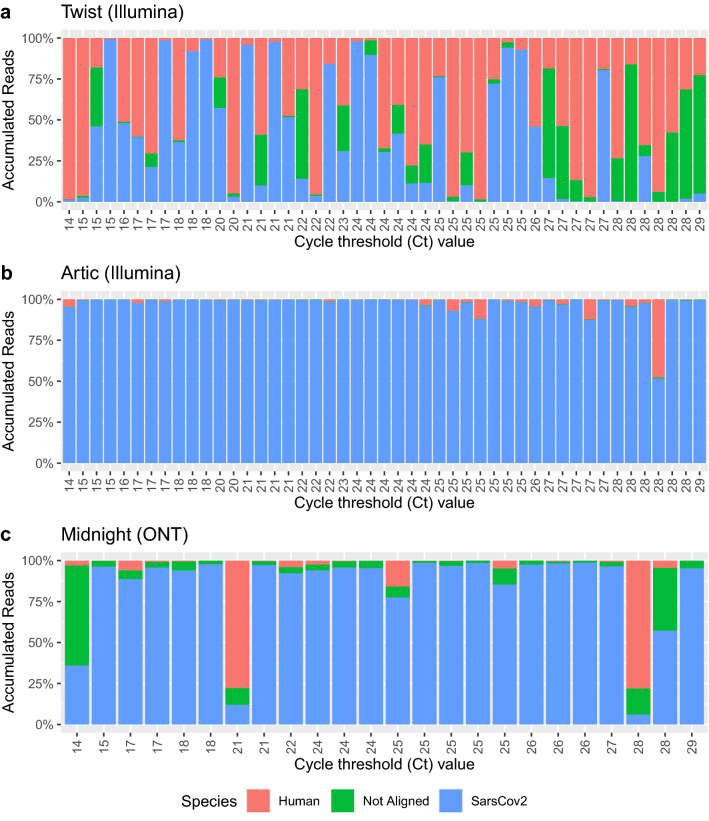
Human host content. Cycle threshold value versus number of raw reads in per cent mapped to hg19, SARS-CoV-2, or unaligned, using (**a**) Twist bait hybridization enrichment (n = 47), (**b**) ARTIC V3 tiled amplicon enrichment (n = 47), (**c**) Midnight tiled amplicon enrichment (n = 24).

Human background and SARS-CoV-2 capture enrichment efficiency in relation to viral load is shown in Fig. 4. Twist showed the highest host content, with a mean fraction of 0.46 of raw reads mapped to the human host genome reference (hg19). A small tendency toward increasing human reads and discarded unmapped reads was seen in samples with Ct values ≥ 27. Batch A-B had high % of reads mapped to SARS-CoV-2 and almost no reads mapped to human, while batch C-G had lower % of reads mapped to SARS-CoV-2 and more host content (Supplementary Table 1 and Supplementary Fig. 1). Overall, both amplicon-based methods showed high SARS-CoV-2 fraction and low host content, with a few exceptions (Fig. 4a,b). ARTIC showed low host content, with a mean fraction of 0.024 human reads. Midnight had a mean fraction of 0.087 human reads.

### Comparison of complete genome consensus sequences

Pangolin and Nextclade assessment and quality control metrics were generated from consensus sequences of SARS-CoV-2 (Supplementary Table 2). All Pangolin and Nextclade designations were in concordance across methods when reads were sufficient for variant classification. The majority of genetic variants that differed from the Wuhan reference were located in the ORF1ab gene and in the S gene, and consisted of substitutions, a few deletions, and one frameshift. For the Twist protocol, 42/47 samples were classified using Pangolin and 46/47 samples were classified using Nextclade. Unclassified samples all had Ct > 25. For successful variant determination from the Twist protocol, 1.5 M SARS-CoV-2 specific reads were needed. The five unclassifiable samples had < 0.65 M SARS-CoV-2 specific reads, and quality control status was bad or mediocre (indicated by “qc.overall” according to Nextclade in Supplementary Table 2). Pangolin and Nextclade successfully classified all 47/47 ARTIC samples. Patient ID 17, with low viral load (Ct value 28), could be classified using the ARTIC protocol but not with Twist or Midnight. Using the Midnight protocol, Pangolin was able to classify 18/24 samples and Nextclade classified 21/24 samples.

**Figure 5:**
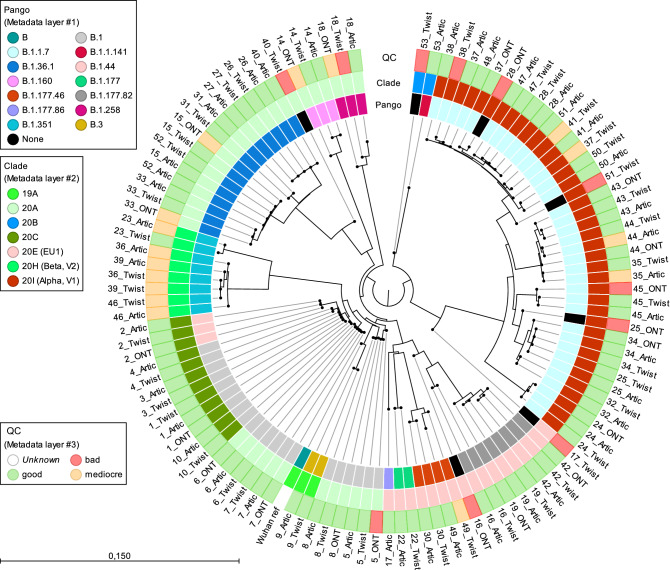
Neighbour joining tree from whole genome comparison of consensus sequences of SARS-CoV-2 genomes generated with Twist bait hybridization enrichment and ARTIC V3 tiled amplicon enrichment on Illumina as well as Midnight tiled amplicon enrichment on Oxford Nanopore Technologies (ONT). Pangolin lineage (Pango), Nextclade (Clade) and Nextclade quality control (QC) measures to flag potential problematic sequences, shown in circles.

The phylogeny, Nextclade, and Pangolin lineages for the consensus sequences are shown in Fig. 5. Patient IDs are in chronological order, starting with sample 1 collected on 1 July 2020 and ending with sample 53 collected on 1 April 2021. The dominance of Pango B.1 in the early pandemic (8 patients) corresponds to the Northern Italian outbreak early in 2020, which was dominant in Sweden at the beginning of the pandemic. The other Pango lineages (Fig. 5) were B.1.1.7 (UK lineage/Alpha; 14 patients), B1.36.1 (European/Denmark lineage; 6 patients), 1.351 (South Africa lineage/Beta; 4 patients) and B1.177.82 (Scandinavian lineage; 3 patients), representing the dominant lineages in Sweden during the period of collection. While Twist generated the complete genome, ARTIC and Midnight showed lack of coverage at the start, end and, mid sections of the SARS-CoV-2 Wuhan reference genome. Twist had the most complete coverage in the first ~ 60 bp. Midnight and ARTIC read coverage started at position 55 in the Wuhan reference and ended at position 29,786 of the alignment with Midnight and position 29,854 of the alignment with ARTIC, due to the primer design (Supplementary Fig. 1). Lack of coverage in the mid sections of the SARS-CoV-2 genome, mainly with the Midnight protocol, is indicated by an entry of “bad” in the “qc.missing” column of Supplementary Table 2.

## Discussion

Genomic surveillance of viral material is important for managing viral outbreaks and understanding the evolution of the virus. This study aimed to evaluate and provide insights into methodological differences between hybridization bait capture and tiled amplicon enrichment for SARS-CoV-2 short-read and long-read sequencing.

Our results showed a better coverage uniformity when using Twist (fold-80 base penalty median of 1.63), compared to ARTIC (median 1.81) and Midnight (median 2.08). This is in concordance with previous studies showing that capture-based methods generally have better uniformity^19,20^. Twist also had the most complete coverage, since neither the ARTIC nor the Midnight amplicon design covered the 5’ and 3’ untranslated regions of the genome. Whole genome deep sequencing with coverage of the entire genome could be important for several research applications, such as viral quasispecies analysis. All methods showed a drop in coverage around 21,000–24,000 bp most likely due to primer or probe specificity in this region. Because this region is partially located in the S gene encoding the spike protein, which is subjected to a high level of mutations, it may be of high importance for emerging variants to improve the designs to generate higher coverage of this region. Indeed, several updates have been published for the ARTIC and Midnight protocols since version 3 and version 1 used in this study, respectively. According to previous studies comparing both ARTIC V3 primers with the updated V4 primers as well as Midnight with Midnight v2 primers, showing the same drop-offs as in our study especially relevant for the Omicron variant (not included in our study)^21–23^, the primer updates should address the drop-offs in the region 21,000–24,000 bp observed in this study.

Limitations of the Twist method included the intra-variation of total reads and reads mapped to SARS-CoV-2 within each batch, which varied the most for Twist independent of Ct values; samples with equal numbers of total reads had a difference of 40–50% in reads mapped to SARS-CoV-2. Similar wide range of target yield of mapped reads for Twist has been shown in previous studies^24^. This is probably an effect of the low RNA input; the Twist method requires an input of 50 ng RNA, but many of the RNA samples were too low to measure. Capture-based methods generally require relatively large inputs of nucleic acid^25^. The between-batch variation in the fraction of reads mapped to SARS-CoV-2 was also the largest with Twist, possibly due to the Twist method being more sensitive to handling and RNA degradation, which lead to a high variation in human host reads. Another disadvantage of the capture-based method is the more costly and laborious workflow.

In concordance with previous studies^19^, we found that amplicon-based methods had higher on-target rates. In our study, the ARTIC amplicon-based target enrichment approach with Illumina short-read sequencing showed the most stable distribution of total reads among samples and the highest percentage of reads mapped to SARS-CoV-2. Meanwhile, the Midnight amplicon-based target enrichment and long-read sequencing with ONT was simple and rapid, and this method is more cost-efficient for fewer samples. ONT sequencing was run for 12 h, optimized to meet the ONT recommendations based on > 20,000 reads for an even genome coverage of SARS-CoV-2^9^. This recommendation is in alignment with our results, samples with less than 20,000 reads could not generate lineage designations. Another three samples had > 20,000 reads but were assigned as bad quality according to Nextclade, and could not be assigned a lineage. This could be due to high Ct values in two of the samples (Ct 27 and 28), which could have affected the coverage uniformity. In addition, two Midnight samples with Ct < 25 had < 50% of mapped reads to SARS-CoV-2. One (Ct 21) had been diluted and the other (Ct 14) had low specific reads for the Twist method as well, indicating that some samples are harder to sequence, possibly due to pre-analytical factors.

Inherent differences in the methods may have influenced the comparison. For example, the amplicon-based protocols included separately enriched, normalized, and pooled samples before sequencing the final library, which contributed to a more even sequencing depth between samples. Hybridization-based capture samples using Twist were pooled before enrichment and then amplified in a post-capture PCR before sequencing the final library, and therefore could not be individually normalized before sequencing. Moreover, the amplicon-based protocols specified the input material based on Ct value, which gave a more even viral load among samples and subsequently a more even sequencing depth.

The present study has several limitations. Not all of the patient samples compared with the Twist and ARTIC method could be compared to the Midnight protocol, as this study was conducted in a clinical setting where some samples could not be recovered. Indeed, 7 out of 24 samples analysed with the Midnight method did not have sufficient volume, and had to be diluted (batch X), though this did not have large effect on the overall quality measures. Another 8 out of 24 samples had no RNA extraction left after being run with Twist and ARTIC and therefore had to be re-extracted, which yielded higher Ct values and made those samples difficult to compare with the other methods. Because only 24 out of 47 samples were run with the Midnight protocol, this method was under sampled and this may have led to a potential bias in the analysis. Also, because all three methods were not run in parallel, it is possible that the methods which are less sensitive to RNA degradation performed better.

In conclusion, investigating the advantages and limitations of different protocols is important for population-level WGS of SARS-CoV-2. The methods evaluated in the present study have different strengths and weaknesses, and can suit different clinical and research purposes. Twist can be used to study the entire genome with good coverage uniformity. ARTIC is a robust method that fits well in a more clinical environment, and Midnight and ONT sequencing has superior portability and sequencing time. Comparative studies on new methods that have been published or are under development are needed in order to assess performance and practical applicability.

## Data availability

Data supporting the results are available as assembled FASTA consensus sequences at GenBank with submission-ID: SUB12123746 and accession-ID: OP585690-OP585783.

## Supplementary Information

Supplementary Information.
