# Supplementary material for: Comparison of SARS-CoV-2 whole genome sequencing using tiled amplicon enrichment and bait hybridization
Source: Sci Rep. 2023 Apr 20;13:6461. doi: 10.1038/s41598-023-33168-1 (PMC10116481; doi:10.1038/s41598-023-33168-1)
Supplement: Supplementary file 1 — Supplementary Information. [file 41598_2023_33168_MOESM1_ESM.pdf]

Title: Comparison of SARS-CoV-2 whole genome sequencing using tiled amplicon enrichment and bait hybridization

Anita Koskela von Sydow, PhD[1,2,3,*], Carl Mårten Lindqvist, PhD[2,3], Naveed Asghar, PhD[2], Magnus Johansson PhD[2], Martin Sundqvist, MD PhD[4], Paula Mölling, PhD[3,4], and Bianca Stenmark, PhD[1,2,3]

[1] Department of Laboratory Medicine, Clinical Pathology and Genetics, Faculty of Medicine and Health, Örebro University, Örebro, Sweden

[2] School of Medical Sciences, Faculty of Medicine and Health, Örebro University, Örebro, Sweden

[3] Clinical Genomics Örebro, Science for Life Laboratory, Faculty of Medicine and Health, Örebro University, Örebro, Sweden

[4] Department of Laboratory Medicine, Clinical Microbiology, Faculty of Medicine and Health, Örebro University, Örebro, Sweden

Supplementary Table 1. Sequencing metrics. Patient ID, Ct value from the RdRp/S gene real-time PCR, total reads, % mapped to SARS-CoV-2, mean depth, fold-80 base penalty and breadth of coverage at depth 1× across methods.

		Twist (Illumina)						ARTIC (Illumina)						Midnight (ONT)						
Ct	ID	Total reads	SARS-CoV-2 %	Mean depth	Fold-80	Breadth of coverage %	Batch	Total reads	SARS-CoV-2 %	Mean depth	Fold-80	Breadth of coverage %	Batch	Ct	Total reads	SARS-CoV-2 %	Mean depth	Fold-80	Breadth of coverage %	Batch
14	22	1,924,208	1	47	2.13	100	D	748,582	96	2,872	2.36	100	L	14	8,622	35	18	2.03	99	Y
15	23	1,218,298	3	67	1.28	100	D	825,796	100	3,273	2.45	99	L	n/a	n/a	n/a	n/a	n/a	n/a	n/a
15	24	5,640,558	46	5,679	1.37	100	D	586,536	100	2,207	1.65	100	M	26[b]	188,824	96	1,857	2.31	100	Z
15	6	15,606,562	99	34,929	1.55	100	B	674,612	100	2,620	1.52	100	J	15[a]	116,896	94	1,049	1.67	100	X
16	25	4,864,862	48	5,105	1.45	100	D	599,468	100	2,241	1.74	100	M	27[b]	74,918	96	637	n/a	74	Z
17	26	3,751,408	39	3,160	1.47	100	D	734,496	100	2,864	1.66	99	L	n/a	n/a	n/a	n/a	n/a	n/a	n/a
17	19	9,454,758	21	4,349	2.22	100	C	1,302,184	98	4,611	1.53	100	K	17	580,353	89	3,263	2.39	100	Y
17	7	4,554,064	99	10,114	1.56	100	B	651,524	99	2,533	1.54	100	J	17[a]	88,330	94	821	1.66	100	X
18	28	1,651,548	36	1,231	1.87	100	D	790,162	100	2,985	1.82	100	L	18	82,984	93	565	14.13	99	Y
18	27	26,509,100	92	53,532	1.32	100	D	682,864	100	2,708	1.55	100	L	n/a	n/a	n/a	n/a	n/a	n/a	n/a
18	1	23,049,556	99	51,181	1.40	100	A	706,156	100	2,732	1.49	100	J	18[a]	108,493	96	1,040	1.62	100	X
20	31	13,556,078	57	17,495	1.25	100	E	747,452	100	2,891	1.69	100	L	n/a	n/a	n/a	n/a	n/a	n/a	n/a
20	30	2,322,628	3	155	1.44	100	E	741,246	100	2,910	2.20	100	L	n/a	n/a	n/a	n/a	n/a	n/a	n/a
21	9	752,226	96	1,630	1.45	100	B	597,534	100	2,332	1.58	100	J	21[a]	11,839	15	12	n/a	57	X
21	32	6,076,800	10	1,363	1.41	100	E	631,188	100	2,278	1.73	100	M	n/a	n/a	n/a	n/a	n/a	n/a	n/a
21	8	1,104,794	98	2,417	1.65	100	B	479,452	100	1,442	1.83	100	J	21[a]	79,307	96	691	1.79	100	X
21	33	10,320,780	52	11,681	1.27	100	E	572,818	100	2,075	1.51	100	M	25[b]	179,510	97	1,869	1.78	99	Z
22	34	15,393,650	14	4,869	1.33	100	E	623,694	100	2,295	1.65	100	M	25[b]	179,538	95	1,716	2.38	99	Z
22	35	2,592,334	4	203	1.43	100	E	624,642	100	2,257	2.05	99	M	n/a	n/a	n/a	n/a	n/a	n/a	n/a
22	5	143,354	84	270	1.78	100	B	667,716	99	2,636	1.76	100	J	22[a]	21,818	92	177	1.77	99	X
23	36	6,761,642	31	4,737	1.25	100	E	756,708	100	2,876	1.87	99	L	n/a	n/a	n/a	n/a	n/a	n/a	n/a
24	2	547,892	98	1,186	1.70	100	A	670,528	100	2,636	1.75	100	J	24[a]	57,717	93	496	2.00	99	X
24	39	11,251,774	90	23,199	1.42	100	F	794,460	100	3,142	1.77	99	L	n/a	n/a	n/a	n/a	n/a	n/a	n/a

24	37	3,494,144	30	2,317	1.41	100	E	808,300	100	3,065	2.08	100	L	24	319,256	94	2,032	2.38	99	Y
24	38	1,436,834	41	1,331	1.89	100	F	802,532	100	2,866	3.47	100	L	n/a	n/a	n/a	n/a	n/a	n/a	n/a
24	15	9,149,422	12	2,332	1.34	100	C	1,004,222	100	3,792	1.66	100	K	24	491,183	94	3,322	1.94	99	Y
24	45	821,442	12	211	1.98	100	F	173,138	97	685	2.00	100	L	25[b]	66,842	85	569	6.70	99	Z
25	41	3,514,144	76	6,135	1.64	100	F	817,218	100	3,066	2.71	100	L	n/a	n/a	n/a	n/a	n/a	n/a	n/a
25	18	5,553,528	0	25	1.94	100	C	1,347,520	93	4,464	2.16	100	K	25	114,683	79	719	2.13	99	Y
25	44	1,310,444	10	303	1.76	100	F	625,026	98	2,102	1.66	100	M	26[b]	128,254	96	1,386	2.20	99	Z
25	40	1,050,112	0	9	2.15	99	F	682,814	90	2,329	3.40	100	L	n/a	n/a	n/a	n/a	n/a	n/a	n/a
25	42	3,656,174	72	6,017	1.38	100	F	726,470	99	2,888	1.70	100	L	25[b]	137,433	97	1,477	1.61	99	Z
25	43	28,273,804	94	60,105	1.40	100	F	529,042	100	1,922	1.60	99	M	26[b]	148,513	97	1,620	2.27	100	Z
25	4	85,464	93	178	1.81	100	A	626,888	98	2,429	1.73	100	J	n/a	n/a	n/a	n/a	n/a	n/a	n/a
26	10	33,786	46	35	1.59	100	B	737,660	96	2,724	1.88	100	J	n/a	n/a	n/a	n/a	n/a	n/a	n/a
27	46	8,493,786	14	2,750	1.44	100	G	701,920	100	2,822	1.81	99	L	n/a	n/a	n/a	n/a	n/a	n/a	n/a
27	47	5,455,152	2	193	1.79	100	G	808,702	99	3,173	3.08	100	L	n/a	n/a	n/a	n/a	n/a	n/a	n/a
27	49	5,096,706	0	32	2.13	100	G	793,214	97	2,656	4.66	99	M	n/a	n/a	n/a	n/a	n/a	n/a	n/a
27	48	5,602,436	0	4	n/a	71	G	801,272	88	2,720	3.89	100	L	n/a	n/a	n/a	n/a	n/a	n/a	n/a
27	3	111,370	80	203	1.38	100	A	105,198	99	381	1.65	100	J	n/a	n/a	n/a	n/a	n/a	n/a	n/a
28	51	5,566,040	0	11	1.82	100	G	703,488	98	2,539	3.06	100	L	n/a	n/a	n/a	n/a	n/a	n/a	n/a
28	16	6,795,694	0	69	1.82	100	C	1,083,022	96	3,386	3.17	100	K	28	34,384	57	135	9.00	99	Y
28	50	8,558,300	28	5,360	2.44	100	G	769,488	100	2,809	4.12	100	L	n/a	n/a	n/a	n/a	n/a	n/a	n/a
28	17	7,556,502	0	4	n/a	79	C	1,444,118	55	2,548	3.18	100	K	28	77,698	8	42	3.00	99	Y
28	53	12,254,580	0	10	2.43	95	G	962,766	100	3,041	4.15	99	M	n/a	n/a	n/a	n/a	n/a	n/a	n/a
28	52	8,111,712	2	319	1.40	100	G	730,074	100	2,870	2.70	100	L	n/a	n/a	n/a	n/a	n/a	n/a	n/a
29	14	10,362,824	5	1,145	1.22	100	C	1,033,258	100	3,764	1.81	100	K	29	535,491	94	3,248	1.99	99	Y

Ct, cycle threshold value from the RdRp/S gene real-time PCR; n/a, not applicable.
[a] Original sample diluted, [b] new extraction with new Ct value.

Supplementary Table 2. SARS-CoV-2 clade and lineage assignments. Patient ID, Ct value from RdRp/S gene real-time PCR, Pangolin lineage (Pango), Nextstrain clade (Clade) and quality metrics (according to Nextclade) for Twist bait hybridization enrichment and ARTIC V3 and Midnight tiled amplicon enrichment.

		Twist (Illumina)				ARTIC (Illumina)				Midnight (ONT)				
Ct	ID	Pango	Clade	qc.overall	qc.missing	Pango	Clade	qc.overall	qc.missing	Ct	Pango	Clade	qc.overall	qc.missing
14	22	B.1.177	20E (EU1)	good	good	B.1.177	20E (EU1)	good	good	14	None	None	None	None
15	23	B.1.351	20H (Beta, V2)	good	good	B.1.351	20H (Beta, V2)	mediocre	good	n/a	n/a	n/a	n/a	n/a
15	24	B.1.1.7	20I (Alpha, V1)	good	good	B.1.1.7	20I (Alpha, V1)	good	good	26[b]	B.1.1.7	20I (Alpha, V1)	good	good
15	6	B.1	20A	good	good	B.1	20A	good	good	15[a]	B.1	20A	good	good
16	25	B.1.1.7	20I (Alpha, V1)	good	good	B.1.1.7	20I (Alpha, V1)	good	good	27[b]	None	20I (Alpha, V1)	bad	bad
17	26	B.1.36.1	20A	good	good	B.1.36.1	20A	good	good	n/a	n/a	n/a	n/a	n/a
17	19	B.1.177.82	20E (EU1)	good	good	B.1.177.82	20E (EU1)	good	good	17	B.1.177.82	20E (EU1)	good	good
17	7	B.1	20A	good	good	B.1	20A	good	good	17[a]	B.1	20A	good	good
18	28	B.1.1.7	20I (Alpha, V1)	good	good	B.1.1.7	20I (Alpha, V1)	good	good	18	None	20I (Alpha, V1)	bad	bad
18	27	B.1.36.1	20A	good	good	B.1.36.1	20A	good	good	n/a	n/a	n/a	n/a	n/a
18	1	B.1	20C	good	good	B.1	20C	good	good	18[a]	B.1	20C	good	good
20	31	B.1.36.1	20A	good	good	B.1.36.1	20A	good	good	n/a	n/a	n/a	n/a	n/a
20	30	B.1.177.46	20E (EU1)	good	good	B.1.177.46	20E (EU1)	good	good	n/a	n/a	n/a	n/a	n/a
21	9	B.3	19A	good	good	B.3	19A	good	good	21[a]	None	None	None	None
21	32	B.1.1.7	20I (Alpha, V1)	good	good	B.1.1.7	20I (Alpha, V1)	good	good	n/a	n/a	n/a	n/a	n/a
21	8	B.1	20A	good	good	B.1	20A	good	good	21[a]	B.1	20A	good	good
21	33	B.1.36.1	20A	good	good	B.1.36.1	20A	good	good	25[b]	B.1.36.1	20A	mediocre	good
22	34	B.1.1.7	20I (Alpha, V1)	good	good	B.1.1.7	20I (Alpha, V1)	good	good	25[b]	B.1.1.7	20I (Alpha, V1)	good	good
22	35	B.1.1.7	20I (Alpha, V1)	good	good	B.1.1.7	20I (Alpha, V1)	mediocre	good	n/a	n/a	n/a	n/a	n/a
22	5	B.1	20A	good	good	B.1	20A	good	good	22[a]	B.1	20A	bad	bad
23	36	B.1.351	20H (Beta, V2)	mediocre	good	B.1.351	20H (Beta, V2)	mediocre	good	n/a	n/a	n/a	n/a	n/a
24	2	B.1.44	20C	good	good	B.1.44	20C	good	good	24[a]	B.1.44	20C	good	mediocre
24	39	B.1.351	20H (Beta, V2)	mediocre	good	B.1.351	20H (Beta, V2)	mediocre	good	n/a	n/a	n/a	n/a	n/a
24	37	B.1.1.7	20I (Alpha, V1)	mediocre	good	B.1.1.7	20I (Alpha, V1)	good	good	24	B.1.1.7	20I (Alpha, V1)	good	good

24	38	B.1.1.7	20I (Alpha, V1)	bad	good	B.1.1.7	20I (Alpha, V1)	good	good	n/a	n/a	n/a	n/a	n/a
24	15	B.1.36.1	20A	good	good	B.1.36.1	20A	good	good	24	B.1.36.1	20A	mediocre	good
24	45	B.1.1.7	20I (Alpha, V1)	good	good	B.1.1.7	20I (Alpha, V1)	good	good	25[b]	B.1.1.7	20I (Alpha, V1)	bad	bad
25	41	B.1.1.7	20I (Alpha, V1)	mediocre	good	B.1.1.7	20I (Alpha, V1)	good	good	n/a	n/a	n/a	n/a	n/a
25	18	B.1.258	20A	bad	mediocre	B.1.258	20A	good	good	25	B.1.258	20A	mediocre	mediocre
25	44	B.1.1.7	20I (Alpha, V1)	good	good	B.1.1.7	20I (Alpha, V1)	mediocre	good	26[b]	B.1.1.7	20I (Alpha, V1)	good	good
25	40	None	20A	bad	bad	B.1.36.1	20A	good	good	n/a	n/a	n/a	n/a	n/a
25	42	B.1.177.82	20E (EU1)	good	good	B.1.177.82	20E (EU1)	good	good	25[b]	B.1.177.82	20E (EU1)	good	good
25	43	B.1.1.7	20I (Alpha, V1)	good	good	B.1.1.7	20I (Alpha, V1)	good	good	26[b]	B.1.1.7	20I (Alpha, V1)	good	good
25	4	B.1	20C	good	good	B.1	20C	good	good	n/a	n/a	n/a	n/a	n/a
26	10	B.1	20C	good	good	B.1	20C	good	good	n/a	n/a	n/a	n/a	n/a
27	46	B.1.351	20H (Beta, V2)	mediocre	good	B.1.351	20H (Beta, V2)	mediocre	good	n/a	n/a	n/a	n/a	n/a
27	47	B.1.1.7	20I (Alpha, V1)	good	good	B.1.1.7	20I (Alpha, V1)	good	good	n/a	n/a	n/a	n/a	n/a
27	49	B.1.177.46	20E (EU1)	mediocre	mediocre	B.1.177.46	20E (EU1)	good	good	n/a	n/a	n/a	n/a	n/a
27	48	None	None	None	None	B.1.1.7	20I (Alpha, V1)	good	good	n/a	n/a	n/a	n/a	n/a
27	3	B.1	20C	good	good	B.1	20C	good	good	n/a	n/a	n/a	n/a	n/a
28	51	None	20I (Alpha, V1)	bad	bad	B.1.1.7	20I (Alpha, V1)	good	good	n/a	n/a	n/a	n/a	n/a
28	16	B.1.177.82	20E (EU1)	good	good	B.1.177.82	20E (EU1)	good	good	28	None	20E (EU1)	bad	bad
28	50	B.1.1.7	20I (Alpha, V1)	good	good	B.1.1.7	20I (Alpha, V1)	good	good	n/a	n/a	n/a	n/a	n/a
28	17	None	20E (EU1)	bad	bad	B.1.177.86	20E (EU1)	good	good	28	None	None	None	None
28	53	None	20B	bad	bad	B.1.1.141	20B	good	good	n/a	n/a	n/a	n/a	n/a
28	52	B.1.36.1	20A	good	good	B.1.36.1	20A	good	good	n/a	n/a	n/a	n/a	n/a
29	14	B.1.160	20A	good	good	B.1.160	20A	good	good	29	B.1.160	20A	mediocre	mediocre

Ct, cycle threshold value from the RdRp/S gene real-time PCR; n/a, not applicable.
[a] Original sample diluted, [b] new extraction with new Ct value.

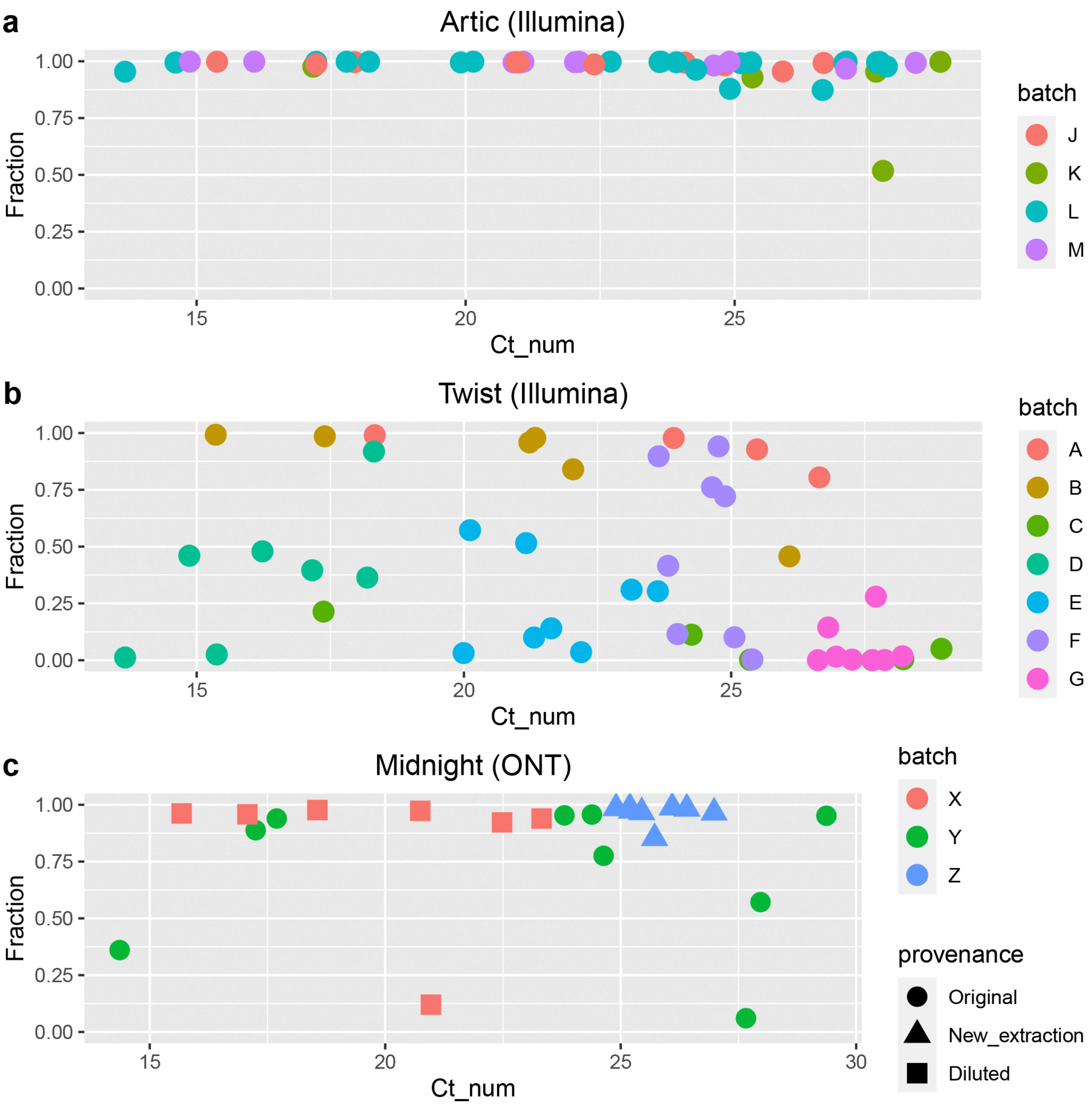

Supplementary Figure 1. Fraction of reads mapped to SARS-CoV-2 versus Ct values. a) Twist bait hybridization enrichment samples sequenced in seven batches, A–G. b) ARTIC V3 tiled amplicon enrichment samples sequenced in four batches, J–M. c) Midnight tiled amplicon enrichment samples sequenced in four batches, X–Z. Sample provenance is indicated: original extraction, diluted original extraction, or new extraction.

a

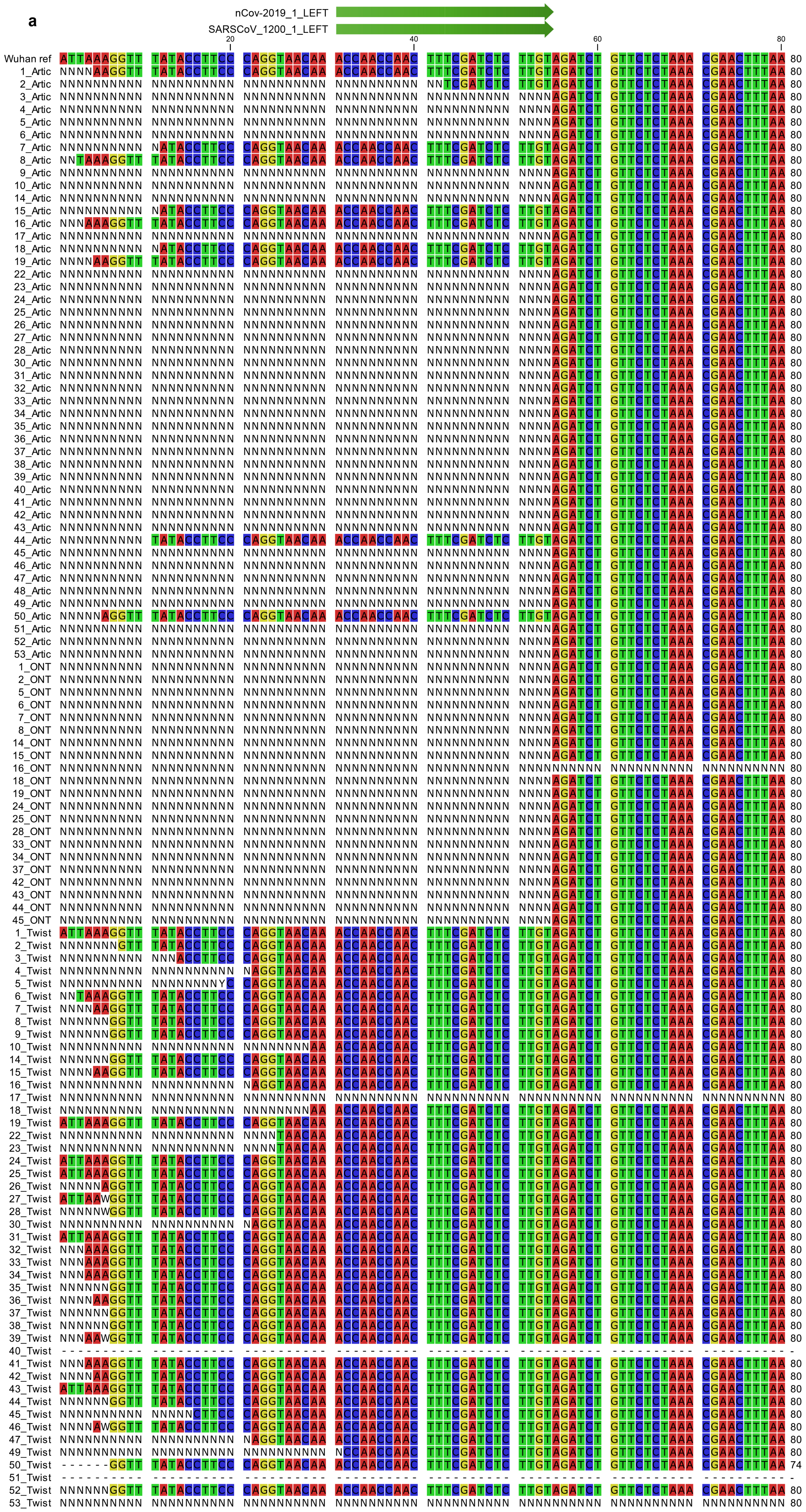
nCov-2019_1_LEFT
SARSCoV_1200_1_LEFT
20
40
60
80
Wuhan ref
1_Artic
2_Artic
3_Artic
4_Artic
5_Artic
6_Artic
7_Artic
8_Artic
9_Artic
10_Artic
14_Artic
15_Artic
16_Artic
17_Artic
18_Artic
19_Artic
22_Artic
23_Artic
24_Artic
25_Artic
26_Artic
27_Artic
28_Artic
30_Artic
31_Artic
32_Artic
33_Artic
34_Artic
35_Artic
36_Artic
37_Artic
38_Artic
39_Artic
40_Artic
41_Artic
42_Artic
43_Artic
44_Artic
45_Artic
46_Artic
47_Artic
48_Artic
49_Artic
50_Artic
51_Artic
52_Artic
53_Artic
1_ONT
2_ONT
5_ONT
6_ONT
7_ONT
8_ONT
14_ONT
15_ONT
16_ONT
18_ONT
19_ONT
24_ONT
25_ONT
28_ONT
33_ONT
34_ONT
37_ONT
42_ONT
43_ONT
44_ONT
45_ONT
1_Twist
2_Twist
3_Twist
4_Twist
5_Twist
6_Twist
7_Twist
8_Twist
9_Twist
10_Twist
14_Twist
15_Twist
16_Twist
17_Twist
18_Twist
19_Twist
22_Twist
23_Twist
24_Twist
25_Twist
26_Twist
27_Twist
28_Twist
30_Twist
31_Twist
32_Twist
33_Twist
34_Twist
35_Twist
36_Twist
37_Twist
38_Twist
39_Twist
40_Twist
41_Twist
42_Twist
43_Twist
44_Twist
45_Twist
46_Twist
47_Twist
49_Twist
50_Twist
51_Twist
52_Twist
53_Twist

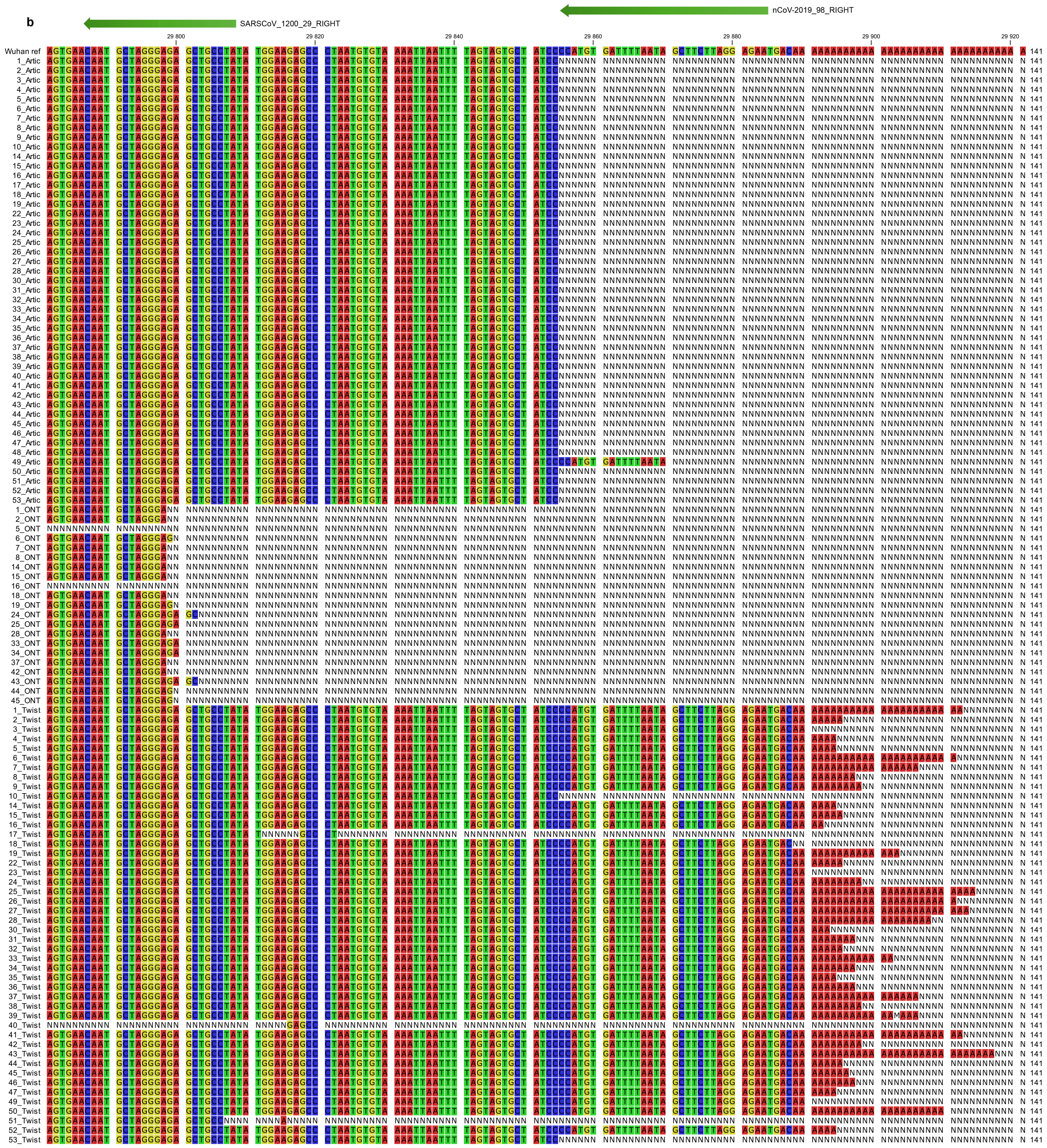

Supplementary Figure 2. a) First 80 bp of the SARS-CoV-2 consensus start. b) Last 141 bp of the SARS-CoV-2 consensus end. Wuhan SARS-CoV-2 reference in first row followed by aligned genomes using ARTIC, Midnight (ONT), and Twist protocol. “Ns” indicates missing sequences in the consensus sequences of the alignments. ARTIC (nCov-2019) and Midnight (SARSCoV_1200) primers shown with arrows at the top.
